# The content validity of the PSS in patients with plaque psoriasis

**DOI:** 10.1186/s41687-017-0004-7

**Published:** 2017-09-12

**Authors:** A. M. Rentz, A. M. Skalicky, K. Burslem, K. Becker, D. Kaschinski, D. Esser, D. A. Revicki

**Affiliations:** 1 Evidera, 7101 Wisconsin Avenue, Suite 1400, Bethesda, MD 20814 USA; 2 Evidera, Seattle, WA USA; 3 Boehringer Ingelheim GmbH, Ingelheim am Rhein, Germany

**Keywords:** Plaque psoriasis, Symptoms, PSS, Pro

## Abstract

**Background:**

The primary objective of this study was to evaluate the content validity of the Psoriasis Symptom Scale (PSS), with a specific focus on understanding of the content of the PRO measure by conducting one-on-one interviews with patients with moderate to severe plaque psoriasis. This was a cross-sectional, qualitative study conducted with 20 patients with plaque psoriasis who participated in in-person, one-on-one interviews. Participants were asked to describe their psoriasis symptoms, completed the PSS, and were cognitively debriefed on its content. Interviews were conducted in two separate rounds. Following Round 1, the study data were examined to determine if modifications to the PSS were required. All interviews were audio-recorded and transcribed. Sociodemographic and clinical data were collected for sample descriptive purposes.

**Results:**

The 20 study participants had a mean age of 50.2 ± 12.0 years (range: 25.0–73.0), and 55% were female. Thirty-five percent of the sample reported their psoriasis severity as moderate or severe. The average time since diagnosis of plaque psoriasis was almost 18 years, ranging from less than one to over 38 years. The most frequently reported symptoms and signs during the concept elicitation portion of the interviews included redness (*N* = 20, 100%), itching (*n* = 20, 100%), pain (*n* = 15, 75%), burning (*n* = 13, 65%), and flaking (*n* = 11, 55%). Overall, participants provided positive feedback on the PSS and felt that it was comprehensive and relevant to their experience with psoriasis. The item meaning and response options were well-understood for the majority of the items. Findings indicate that for the patient-reported symptom of redness, which is also a sign that can be reported by clinicians, redness or the perception of redness is most accurately captured by patient report. Study results did not support modifications to the instrument and no changes to the PSS were recommended.

**Conclusion:**

The evidence gained in this study provided support for the content validity of the PSS for use as clinical trial endpoint among patients with plaque psoriasis. This study found that the symptoms included in the PSS are important to and well-understood by patients with plaque psoriasis. The PSS is appropriate for inclusion in future studies designed to measure the effect of treatment on psoriasis-related symptoms.

## Background

Psoriasis is a lifelong chronic inflammatory disease affecting 2–3% of the worldwide population. An estimated 7.4 million US adults were affected with psoriasis in 2013. The prevalence of psoriasis amongst US adults 20 years and older is 3.2% (95% confidence interval [CI] 2.6%–3.7%) with Caucasians reporting the highest prevalence (3.6%) [1]. Plaque psoriasis is the most common clinical form and is characterized by red, itchy, scaly and painful plaques generally localized on the scalp, elbows and knees [2]. Major determinants of psoriasis severity include the extent of skin involvement; localization in highly affected areas such as scalp, palms, and soles; pruritus; presence of comorbidities including psoriatic arthritis; and impairment of a patients’ emotional, social, occupational and physical functioning [3, 4].

Clinical trials evaluating the signs and symptoms of psoriasis treatment often incorporate self-reports from patients, commonly referred to as patient-reported outcomes (PRO). PROs provide important information about symptoms associated with psoriasis or its treatment such as pain, itching, and burning which can only be evaluated or assessed by the patient; not through clinical assessment or testing. Symptoms are considered to be something that a patient observes, while signs are observable by a clinician or another person. In some cases signs may often be assessed more appropriately by clinicians using clinical assessments. It is possible, however, for something to be both a symptom and a sign: evaluable by both a patient and a clinician.

A PRO measure that includes symptoms of psoriasis, and not signs of psoriasis, is lacking. To meet this need, the Psoriasis Symptom Scale (PSS) measure was developed. The measure was developed through a review of common symptoms associated with psoriasis. In addition, two PRO psoriasis measures were found and reviewed: the Psoriasis Symptom Index (PSI) [5] and the Psoriasis Symptom Diary (PSD) [6]. The PSI contains eight symptoms and signs of psoriasis, including itchiness, redness, scaling, burning, cracking, stinging, flaking and pain. The PSD includes 20 items encompassing symptom severity, bother, and functional impacts of itch, stinging, burning, cracking, pain, scaling, and skin color change. The PSS was developed through an iterative process of literature review, clinical expert and PRO expert review, and FDA advice on PRO implementation. From the literature and instrument review, four items were determined to be the key indicators of severity of plaque psoriasis: pain, itch, burning, and redness. The inclusion of redness, which may be considered both a symptom and a sign, was determined to be an item that may be more accurately reported by patient-report rather than clinician-report because patients may interpret “redness” or their perception of redness of their own skin differently than clinicians. A recent study supports that although the term “redness” is included in clinical assessments, qualitative data indicates that the consistency of lesion color are highly variable among patients, especially among patients with darker skin tones, yet “redness” is highly relevant to patients but may be difficult for clinicians to accurately report [7].

Per the Food and Drug Administration’s (FDA) guidance on the development and use of PROs, new PRO measures must be evaluated for content [8]. Content validity of a PRO measure requires conducting interviews with patients with the condition of interest to ensure that an instrument’s instructions are clear, the content of each question is appropriate and understandable from the participant’s perspective, and that the intended meaning of each item is consistent with the participant’s interpretation or assigned meaning [9]. Understanding of the response scales and recall period is also evaluated.

The primary objective of the current study was to evaluate the content validity and patient interpretation of the PSS with patients with moderate to severe plaque psoriasis.

## Methods

### Study design

This was a qualitative study involving participants with plaque psoriasis who took part in semi-structured, in-person interviews. The in-depth interview involved two parts: 1) concept elicitation of psoriasis-related symptoms during which participants talk about their psoriasis symptom experience; and 2) cognitive debriefing of the PSS during which participants’ comprehension and interpretation of the items, recall period and response options is assessed. The methods used in this study followed the FDA guidance on the use and development of PROs and clinical outcome assessments to support efficacy and labeling claims [8, 10].

Participants were recruited from three dermatology clinics in Arkansas, Massachusetts, and Virginia using a standardized screening script. Site study staff identified eligible participants from patient databases, charts, and/or daily appointment schedules. All recruitment procedures complied with current Health Insurance Portability and Accountability Act of 1996 (HIPAA) regulations and the study was approved by a central institutional review board (IRB). All study participants provided written informed consent for participation in the interview.

Participants who met the following inclusion criteria were invited to participate in the study: adults 18 and older; clinician-confirmed diagnosis of moderate to severe plaque psoriasis; confirmation by clinician that patient is being treated for psoriasis with either conventional (including PUVA) or biologic systemic treatment; and willing to be audio-recorded during the interview session.

Participants were excluded from the study if they were being treated for psoriasis exclusively with topical medication; had a diagnosis of non-plaque forms of psoriasis; were currently participating in a clinical trial being conducted by the study sponsor; had a history of other chronic pain conditions, other dermatologic conditions; or severe psychiatric comorbidity or other impairment (e.g., cognitive, neurological, visual) that would interfere with participating in a one-on-one discussion.

**Fig. 1:**
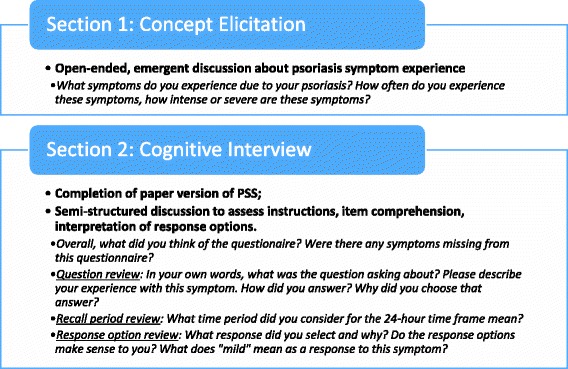
Interview Summary

Participants completed a one-time, in-person, individual, hour-long interview. An experienced qualitative researcher conducted the interviews using a semi-structured cognitive interview discussion guide which was designed to elicit symptom experiences from participants and assess the content validity of the PSS. The interview was audio-recorded. Figure 1 summarizes the interview process. Participants also completed a sociodemographic questionnaire and the site clinician provided information on the participant’s psoriasis history, treatments and comorbidities.

### Psoriasis symptom scale (PSS)

The 4-item PSS is designed to measure patient-reported psoriasis symptoms. The PSS was adapted from the literature and previously published psoriasis symptoms scales [5, 6], which were developed with extensive patient input [6, 7]. The PSS consists of four items assessing severity of pain, itching, redness, and burning during the past 24 h. A 5-point severity scale was used as follows: 0 = none, 1 = mild, 2 = moderate, 3 = severe, 4 = very severe. The PSS is hypothesized to sum to an unweighted total score (See Appendix).

### Analyses

Descriptive statistics (mean, standard deviation, frequency) were used to characterize the sample in terms of sociodemographic, clinical characteristics, and PSS results.

A content analysis approach was used to analyze the interview data using ATLAS.ti version 7.1.5 qualitative analysis software. A coding dictionary was developed based on the interview guide. Participant responses were coded by trained researchers to examine comprehension, relevance to their experiences, and ease or difficulty of selecting a response for each item of the PSS. The coding and analysis organized the interview data by interview topic. A proportion of all coded transcripts were reviewed by a second team member. Qualitative findings were summarized with exemplary quotes as well as frequencies and percentages as appropriate.

## Results

A total of 20 interviews were conducted in January 2016 and took place in three sites located in the US: Arkansas (30%, *n* = 6), Massachusetts (25%, *n* = 5), and Virginia (45%, *n* = 9). Complete sociodemographic characteristics for the study sample are presented in Table 1. Mean age was 50.2 ± 12.0 years (range: 25.0–73.0), with 55% (*n* = 11) female, 85% (*n* = 17) white and one Hispanic participant. Almost two-thirds of the participants reported that they worked full-time (*n* = 13, 65%).

**Table 1:** Participant Self-Reported Sociodemographic and Clinical Characteristics

	Total Sample (*N* = 20)
Age: Mean age ± SD	50.2 ± 12.0
Range (Min, Max)	(25.0–73.0)
Female (%)	11 (55%)
Ethnicity: Hispanic or Latino (%)	1 (5%)
Racial background, n (%)^a^	
White	17 (85%)
Black/African American	2 (10%)
Missing^a^	1 (5%)
Current living/domestic situation, n (%)	
Living alone	4 (20%)
Living with partner/family/friend	16 (80%)
Employment status, n (%)	
Employed^b^	17 (85%)
Not-employed^c^	3 (15%)
Highest level of education, n (%)	
Secondary/high school	9 (45%)
Some college	5 (25%)
College degree	6 (30%)
Overall health rating, n (%)	
Excellent	2 (10%)
Very good	7 (35%)
Good	10 (50%)
Fair	1 (5%)
Poor	0 (0%)
Overall assessment of psoriasis severity, n (%)	
No psoriasis	4 (20%)
Very mild	7 (35%)
Mild	2 (10%)
Moderate	5 (25%)
Severe	2 (10%)
Very severe	0 (0%)

^a^One participant stated race as “Other Puerto Rican”.

^b^Includes self, full-, or part-time employed.

^c^Includes retired and disabled

The majority of participants rated their overall health as good to excellent (*n* = 19, 95%). When self-assessing psoriasis severity, almost two-thirds of participants rated it as none to mild (*n* = 13; 65%) suggesting their psoriasis symptoms were well-controlled by medication. The remaining participants reported their psoriasis severity as moderate or severe (*n* = 7, 35%).

Clinician-reported clinical characteristics for the study sample are presented in Table 2. All participants were confirmed to have moderate to severe plaque psoriasis (*n* = 20, 100%). The average time since diagnosis was almost 18 years, ranging from less than one to over 38 years. The mean length of time on systemic treatment was 4.4 ± 4.8 years (range: 0.1–20.0 years) with most participants currently treated with ustekinumab (*n* = 5, 25%), adalimumab (*n* = 4, 20%), or ixekizumab (*n* = 4, 20%).

**Table 2:** Clinician-Reported Participant Characteristics

	Total Sample (*N* = 20)
Time Since Psoriasis Diagnosis, years	
Mean ± SD	17.9 ± 9.9
Median (IQR)	19.6 (11.1–24.1)
Range (Min – Max)	(0.2–38.6)
Time on Systemic Treatment, years	
Mean ± SD	4.4 ± 4.8
Median (IQR)	2.3 (1.4–7.0)
Range (Min – Max)	(0.1–20.0)
Comorbidities, n (%)	
Anxiety	2 (10%)
Depression	2 (10%)
Psoriatic Arthritis	4 (20%)
Other^a^	12 (60%)
No other health conditions	8 (40%)
Current treatments^b^, n (%)	
Secukinumab	1 (5%)
Etanercept	3 (15%)
Folic Acid	1 (5%)
Adalimumab	4 (20%)
Ixekizumab	4 (20%)
Methotrexate	1 (5%)
Ustekinumab	5 (25%)
Ultraviolet B (shortwave) Waves (UVB)	2 (10%)
Other	1 (5%)

^a^Not mutually exclusive; other comorbidities included: atrial fibrillation, diabetes [6], hyperlipidemia [2], hypertension [5], obesity [2], restless legs, hypothyroidism, post-menopausal, peripheral edema.

^b^Not mutually exclusive

### Concept elicitation results

Tables 3 and 4 present symptoms (Table 3) and signs (Table 4) most frequently reported by participants with key quotes for each symptom or sign. Most commonly reported symptoms included itching (*n* = 20, 100%), pain (*n* = 15, 75%), and burning (*n* = 13, 65%).

**Table 3:** Plaque Psoriasis Symptoms

	N	%	Key quotes
Itching	20	100%	001–004: Very, very intense, I guess you’d say. It’s kind of like if you’ve ever had poison ivy or poison oak, it’s similar to that, but it doesn’t stop. It just keeps on itching and burning until you finally claw it enough, then the next thing you know you’re bleeding, gotta get that stopped.
Pain	15	75%	002–005: it hurt—it hurt, you know, um, almost like a toothache until you were able to tend to it and get something to ease the pain, it was there, you know.
Burning	13	65%	Now, burning, describe it to me again? 003–011: Um, it’s a sensation like, um, I suppose it would be similar to having a severe sunburn or any other type of burn, you just feel a heat sensation that is unbelievably hot in a spot on your leg—in my case it was my legs, lower legs.

**Table 4:** Plaque Psoriasis Signs

	N	%	Key quotes
Redness	20	100%	002–004: It starts—yeah, you start with almost like, um, a rosy colored, you know, where it starts and then, oh, within a matter of a few days you start to see the deeper red, and then, of course, it progresses where it gets like a little crusty and then if you let it go it gets like cracking.
Flaking	11	55%	002–004: It’s scaly, it flakes, um, it’s almost like a dead skin would be when it flakes—it’s annoying. In the very beginning, uh, it was very disturbing, but as I said I’ve had it, and there’s no cure basically for it, so I had to learn to, um, adjust to live with it, basically.
Scales	7	35%	003–007: Um, it was just breakouts, lesions all over my skin, just bit red, patchy, dry, scaly skin
Bleeding	6	30%	003–002: Um, I had to put patches on my elbows, because [the bleeding] was really bad. Anywhere that would bend, um, on my buttocks—I would always have blood on my clothes, because it would crack open every time you would sit down, put pressure.

Other, non-symptom aspects of moderate-to-severe plaque psoriasis mentioned by patients included pain from psoriatic arthritis (*n* = 7, 35%), impact on daily life like avoiding school and feeling self-conscious or socially stigmatized.

Itching was experienced by all participants (*n* = 20, 100%) either currently or in the past. For many, it was a constant experience and extremely bothersome and severe. Many found that it was entirely unavoidable at times, and that there would be days where they could not stop scratching. One participant stated that she worked to train her mind not to scratch; that scratching was a mental thing. Some described the itch as similar to an “insect bite” or like “poison ivy.” In many cases, the itching, if not controlled, would lead to other symptoms, including redness, flaking, and bleeding.

Pain was mentioned by 75% of participants (*n* = 15) who described it in several ways. Most experienced pain as a result of itching and flakiness. Several participants described their pain as a “toothache,” a dull pain that was always there and would flare-up on occasion. A few described the pain as a burning or stinging sensation. During flare-ups, participants stated that the pain was extreme and constant. In addition to pain experienced from other symptoms acting up, some attributed their pain to psoriatic arthritis, a comorbid condition. Other psoriasis-related symptoms overlapping with pain included discomfort (*n* = 1, 5%), as well as pain from psoriatic arthritis (*n* = 7, 35%).

Burning was experienced by 65% (*n* = 13) of all participants. Many described burning as a type of pain or a stinging sensation, that their skin would feel “chapped” or “raw.” One participant stated that burning felt like a severe sunburn that would radiate heat. Overall, burning was not something that was constantly felt, but was something that was for the most part, severe. For several participants, burning was a result of an external trigger. For example, some stated that itching, cold weather, or the application of a topical medication would lead to a burning sensation. Another psoriasis symptom overlapping with burning included stinging (*n* = 2, 10%).

Redness was noted by all participants (*n* = 20, 100%), experienced either currently or in the past. Many participants indicated they would experience redness all the time, and that if left untreated it would progress to itching, flaking, and sometimes, cracking and bleeding. Some participants mentioned that the redness was a constant underlying presence to the plaque, that it would surround the white scales of the psoriasis. Redness was described as occurring in different degrees, from a pinkish red to a deep red. Several participants described the redness as extremely embarrassing; something they would work to cover up. Another symptom overlapping with redness was skin irritation (*n* = 2, 10%).

Fifty-five percent (*n* = 11) of participants mentioned flaking. They described it as patches of dried or dead skin falling off the body. Many participants stated it was a constant experience and would result primarily from scratching, but also from clothing or anything rubbing against the skin. Flaking was quite severe for most participants and many mentioned the embarrassment and discomfort involved with leaving flakes behind wherever they went.

Scales or scaling were experienced by 35% (*n* = 7) of participants. These “patches” were described as white, dry, itchy, and irritating. Many participants spoke of flaking and scaling together and considered them to be similar. Scales would be particularly bothersome during outbreaks or flare-ups. Other psoriasis signs overlapping with flaking and scales included visible plaques (*n* = 4, 20%) and dry skin (*n* = 3, 15%).

Six (30%) participants experienced bleeding. While it did not occur frequently, it was considered severe when present. Bleeding typically resulted from over-scratching, rubbing from clothes, and pressure on the skin from sitting or bending legs or arms. Bleeding would occur in spots that were irritated or dry and cracked.

Other psoriasis signs, symptoms or impacts described by participants included social impacts (*n* = 2, 10%), a feeling of inflammation or sunburn (*n* = 1, 5%), white spots (*n* = 1, 5%), and swollenness (*n* = 1, 5%). Most of these problems were mentioned in conjunction with other signs and symptoms.

### PSS cognitive interview results

**Fig. 2:**
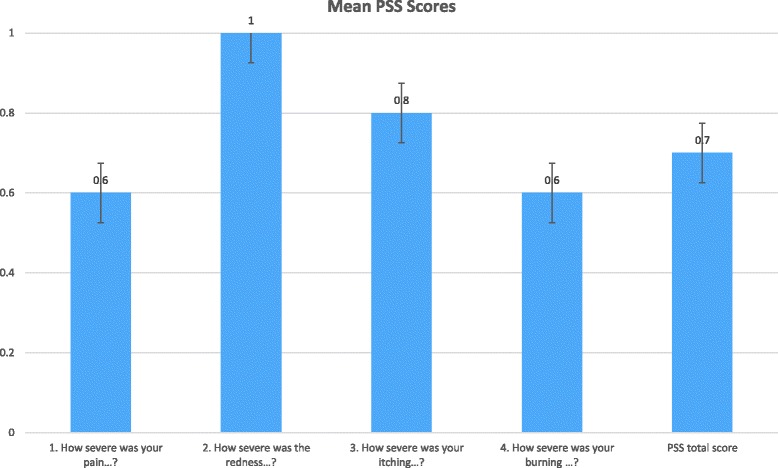
Mean PSS Scores (*n* = 20)

**Fig. 3:**
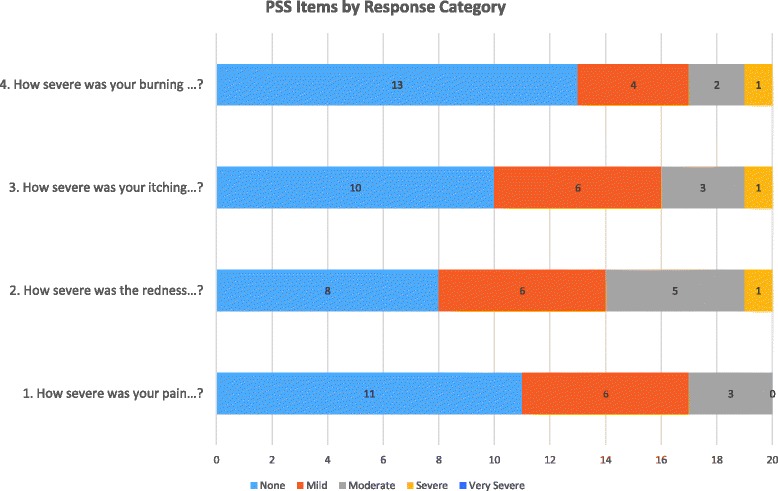
PSS Items

Mean PSS item and total scores are presented in Fig. 2. Response distributions for the PSS items are presented in Table 5 and Fig. 3. In general, participants’ response options ranged from “None” to “Moderate” with only one participant claiming severe symptoms for three of the four items. None of the participants selected the “Very severe” response option.

**Table 5:** PSS Responses

Item	Mean (SD)Median (Range)	Floorn (%)	Ceilingn (%)	None	Mild	Moderate	Severe	Very Severe
1. How severe was your pain…?	0.6 (0.8)0 (0–2)	11 (55%)	0 (0%)	11 (55%)	6 (30%)	3 (15%)	0 (0%)	0 (0%)
2. How severe was the redness…?	1.0 (0.9)1 (0–3)	8 (40%)	0 (0%)	8 (40%)	6 (30%)	5 (25%)	1 (5%)	0 (0%)
3. How severe was your itching…?	0.8 (0.9)1 (0–3)	10 (50%)	0 (0%)	10 (50%)	6 (30%)	3 (15%)	1 (5%)	0 (0%)
4. How severe was your burning…?	0.6 (0.9)0 (0–3)	13 (65%)	0 (0%)	13 (65%)	4 (20%)	2 (10%)	1 (5%)	0 (0%)
PSS total score	0.7 (0.8)1 (0–3)	7 (35%)	0 (0%)	--	--	--	--	--

### Item level feedback

#### Question 1: How severe was your pain due to your psoriasis during the past 24 h?

In general Item 1 was well understood by all 20 participants (100%). When asked to describe the phrase “pain due to your psoriasis” participants used words/phrases such as “tender,” “raw,” “torture,” “sharp,” and “stinging” and talked about pain experiences due to itching, burning, and boils/blisters.

Participants were asked if they would change anything about Item 1, and all but one (*n* = 19; 95%) indicated no changes were necessary. One participant (5%) thought Item 1 could be improved by specifying the type of pain patients should consider when answering the question.

The majority of participants (*n* = 17; 85%) understood the intended timeframe for the question and used the past 24 h when considering their response. Two participants (10%) reported thinking back to when their psoriasis pain was at its worst when selecting a response. Lastly, one participant (5%) considered the previous night when answering the item, but did not specify thinking about the full 24-h timeframe.

Participants generally understood the meaning of the response option “None” to mean there was no pain experienced due to their psoriasis while the response option “Mild” meant there was “some” pain, “a little bit” of pain, or pain “here and there.” Participants said selecting “Moderate” would indicate the severity of their pain had reached the point of worrying about it and/or contacting their doctor. The “Severe” option meant the pain was “worse than moderate,” was more “constant,” and might require treatment to deal with the increased pain. Selecting “Very Severe” for Item 1 would be an indication that a participant’s pain had become “really intense” or “really painful,” to the point where they were unable to function.

#### Question 2: How severe was the redness from your psoriasis during the past 24 h?

All 20 participants (100%) understood the meaning of Item 2 and made no suggestions for improving the item. The majority of participants who were asked to describe the term “redness from your psoriasis” talked in terms of “discoloration,” “red spots or patches,” “inflammation,” and “irritation” of their skin. One participant (5%) suggested that the questionnaire could benefit from the inclusion of a response scale which included skin color to help patients indicate the degree of redness.

Nineteen out of 20 participants (95%) were asked what timeframe they considered when selecting a response and, of those asked, 14 participants (74%) specified that they considered the past 24 h. A couple of participants (*n* = 2; 11%) reported thinking about when their redness was the worst but did not specify a 24-h timeframe. Other participants thought about the present moment, when they got up that morning, or the last two months (*n* = 1 each; 5% for each).

For Item 2, participants interpreted “None” as having their natural skin color with no apparent redness present while “Mild” meant having a pink tint to their skin or having “a little redness.” Most participants said selecting “Moderate” would indicate that their affected skin area had changed from pink to red to a “reddish-pink” or “dark pink” shade. Participants interpreted “Severe” as a change of color from “pinkish-red” to “red” while a “Very Severe” rating would mean the affected skin had turned a shade of “dark red” or “blood red.”

#### Question 3: How severe was your itching from your psoriasis during the past 24 h?

All 20 plaque psoriasis participants (100%) demonstrated an understanding of the itching item. When asked to describe the phrase “itching from your psoriasis,” most participants talked in terms of “scratching,” “rubbing,” and “irritation.” The participants liked the question and did not suggest any changes to improve upon the wording.

Nineteen participants were asked what timeframe they considered when answering Item 3, and all (100%) generally reported basing their answer on their itching experiences within the past 24 h.

Selecting “None” for Item 3 was generally understood by participants to mean there was no itching experienced during the past 24 h due to psoriasis while “Mild” meant their plaques itched “a little bit” or “once in a while” during the past 24 h. Participants said “Moderate” meant they were itching more frequently and the itching continued to come and go through the 24 h. Selecting “Severe” was generally described as itching “all the time” or itching “constantly” while “Very Severe” would mean to most participants that they were constantly or uncontrollably itching and scratching all day and night, to the point where they had scratched off their skin and possibly started to bleed.

#### Question 4: How severe was your burning from your psoriasis during the past 24 h?

All 20 participants (100%) understood Item 4 with no difficulty. The majority of participants described the term “burning from your psoriasis” as “painful,” “raw,” and “stinging.” Participants also defined their burning sensations in terms of heat and used descriptions such as “warm” and “hot.” One participant (5%) understood the question but experienced some difficulty with the response options for this item, saying the question could be improved by collecting a more detailed response on each patient’s subjective experience with burning. Another participant (5%) understood the item, but indicated they would prefer using the word “extreme” for the “very severe” response option.

When asked what time period they had in mind when answering the question, most (*n* = 16; 80%) thought about the past 24 h. A few did not specify the phrase “past 24 h” but correctly reported that they thought about “last night and yesterday during the day,” “since yesterday,” and “all through last night” (*n* = 1 each; 5% for each). One participant (5%) did not report on a timeframe because he had never experienced burning symptoms due to his psoriasis. The remaining three participants (15%) reported thinking about the “last two months until now,” “yesterday and the day before,” or “when the burning was at its worst.”

The absence of burning in the past 24 h would warrant a response of “None” for Item 4 for the majority of participants. A response of “Mild” meant “very little” or “occasional” burning in the past 24 h. Participants generally spoke about selecting “Moderate” when their burning symptoms became more “noticeable” and “bothersome.” They said they would select “Severe” when they were experiencing burning on multiple areas of their body and the burning sensations were becoming more “intense.” Selecting “Very Severe” was reserved for when they experienced “non-stop” or “very painful” burning during the past 24 h.

### Overall comments

Participants were asked to provide their overall impressions of the questionnaire. All 20 (100%) provided positive feedback and responded that the questionnaire and items were “*good*,” “*simple,*” “*thorough,*” and/or “*straightforward.”* They had no difficulties completing it nor did they find anything confusing about it. Overall, participants provided clear explanations for how they responded to the questions and arrived at an answer for all four PSS items.

Participants provided feedback on the relevance of the questionnaire to their plaque psoriasis condition. All participants (*n* = 20; 100%) indicated that the questionnaire content was relevant to their plaque psoriasis symptom experience. One participant (5%) specified that he no longer experiences plaque psoriasis symptoms due to successful treatment, however, the questions would have applied to him when he was still experiencing symptoms. Two participants (10%) commented that the redness and itching questions were applicable to their current condition but the burning and/or pain items were not.

#### Missing items

Participants were asked if there were any concepts missing from the PSS, and several participants (*n* = 7; 35%) reported that the questionnaire was comprehensive and had no suggestions for inclusion of additional items. Other participants suggested incorporating questions to capture social and emotional impacts of plaque psoriasis (*n* = 4; 20%) and signs of flaking (*n* = 4; 20%), scaling (*n* = 3; 15%), peeling (*n* = 1; 5%), and bleeding (*n* = 1; 5%). Lastly, two participants (10%) thought the questionnaire would benefit from inclusion of an item to capture psoriatic arthritis-specific symptoms.

#### Response options

Eighteen out of 20 participants (90%) were asked if they experienced any difficulty selecting a response. Of those, all but one (*n* = 17; 94%) reported that the questions were not difficult to answer. Only one participant (6%) mentioned that she experienced difficulty selecting a response on the redness item because she was not sure what color should be associated with each response option. The majority of participants (*n* = 13; 72%) reported they would not modify the response options and thought the scale worked well with the questions. Other participants suggested removing the “very severe” option, including a descriptive definition for each option, or including room for patient comments (*n* = 1 each; 5% for each).

When asked to provide feedback on the differences between the response options, all participants were generally able to describe clear distinctions between each response option for all four items.

#### Recall period

A few participants (*n* = 3; 15%) offered suggestions related to modifying the questionnaire’s 24-h recall period. One participant (5%) suggested asking patients to rate their severity when thinking about their worst plaque psoriasis experience in addition to asking about the last 24 h. Another participant (5%) reported that the measure would benefit from a 7 or 10-day timeframe to capture changes in symptom severity. Lastly, one participant (5%) mentioned they would prefer each response option be individualized and ask participants about a time when they were experiencing a breakout.

## Discussion

Results from this qualitative study provide support for the content validity of the PSS for use as a clinical trial endpoint among patients with moderate to severe plaque psoriasis. The most frequently reported symptoms of plaque psoriasis by the 20 participants were itching (*n* = 20, 100%), pain (*n* = 15, 75%) and burning (*n* = 13, 65%). The most frequent symptoms/signs were redness (*n* = 20, 100%) and flaking (*n* = 11, 55%).

Overall, participants provided positive feedback on the four items of the PSS and found the measure to be relevant, straight-forward, and easy to understand. The majority of participants said they did not find any aspects of the measure to be confusing. The interviewers probed some participants on their experience with some of the symptoms included in the PSS because the symptoms were not spontaneously mentioned. Most participants then recalled that they did have prior experience with the particular symptom but were not currently experiencing the symptom because their psoriasis was well controlled.

Of particular importance was the participant feedback on the “redness” item, which while both a symptom and sign of psoriasis, appears to be one that is highly subjective and therefore potentially more reliably reportable by patients rather than clinicians. The literature describes that patients with psoriasis experience varying degrees of skin discoloration due to plaque lesions. Studies have explored how participants interpret the “redness” of their skin lesions [7, 11] and the degree to which redness is a clear and important concept experienced by patients with moderate to severe chronic plaque psoriasis. Although the term “redness” is included in clinical assessments, qualitative data supporting consistency of lesion color description are limited, especially among patients with darker skin tones.

Most participants did not have a problem with the 24-h recall period and were able to complete the questionnaire using the appropriate time period. A few participants reported having to think back to a time when they were experiencing the symptom or a flare-up due the effectiveness of current treatment that controls symptoms. Participants also understood and provided clear explanations for their use of the response options associated with the PSS items. Additionally, they were able to provide clear distinctions between each response option for all four items. The majority of participants thought the response scale worked well with the questions and would not modify the response options.

Several participants suggested adding items on flaking or scaling to the PSS. Although many participants had experience with flaking and scaling, these are signs which may be better assessed by clinician-report rather than through patient report. In most clinical trials of plaque psoriasis, the flaking/scaling component of plaque psoriasis is assessed by clinicians using the Psoriasis Area and Severity Index which has been referred to as “the gold standard of severity measurement” [12–15].

Several participants suggested that asking patients about social and emotional impacts of plaque psoriasis would be important. Previous research has demonstrated the impact of psoriasis on patient reported social activities, emotional function, and health-related quality of life [16–19]. While there is a need to assess patients’ health-related quality of life, the developers wanted to keep the PSS limited to symptoms.

Psoriatic arthritis was mentioned by 35% (*n* = 7) of participants, typically when discussing the pain they experienced associated with their psoriasis. For some, psoriatic arthritis was the main source of pain they experienced. For the most part, participants experienced some level of pain or discomfort from their arthritis all of the time, with occasional flare-ups. During flare-ups, the pain would be severe or very severe. Psoriatic arthritis was experienced in the fingers, toes, wrists, and shoulders.

An apparent discrepancy exists in the results between the clinician’s diagnosis of moderate to severe psoriasis and how participants perceived their level of symptom severity at the time of eligibility screening for the study. This discrepancy may be attributed to patients being well-controlled on psoriasis treatment. Clinician report indicated participants had moderate to severe level of plaque psoriasis, however, only 35% of participants self-reported having current moderate to severe disease. Results on the PSS completed by participants were similar; the mean score for the four PSS items ranged from 0.6 to 1.0 on a 0 to 4 scale and almost all participants (*n* = 19) reported “good” or “better” general health score. A possible explanation for these findings may be that all participants were currently being treated with conventional or biologic systemic treatments, and these treatments were keeping participants’ symptoms well controlled leading them to report less severe disease. A number of effective systemic therapies exist for psoriasis that may reduce the number of patients with severe symptoms [20, 21].

This sample may be biased toward participants with well-controlled disease and less severe symptoms of plaque psoriasis. Participants with more severe, active disease may not have been willing to participate in this qualitative research study. Additionally, since effective therapies exist that keep psoriasis symptoms under control, some participants may not have had as many current symptoms as would be expected in the context of a treatment study where disease activity is an inclusion criterion. A limitation of this study is that the participants were predominantly Caucasian. However, Caucasians are disproportionally affected by psoriasis compared to other racial/ethnic groups in the US [1].

## Conclusions

Compared to other similar psoriasis instruments, the PSS instrument was designed to focus solely on symptom severity and symptoms most accurately assessed by patients. Overall, the identification and description of psoriasis-related symptoms are comparable to other qualitative research studies [6, 10]. This research provides evidence that the symptoms included in the PSS are important to and well-understood by patients with plaque psoriasis and the PSS is appropriate for inclusion in studies to measure the effect of treatment for moderate to severe plaque psoriasis.

## Appendix. Psoriasis Symptom Scale

### PSORIASIS SYMPTOM SCALE (PSS)

Listed below is a set of problems that people with psoriasis have said are important. For each question, click on the circle that best describes the severity of your symptoms during the past 24 h. Please answer every question.

1. How severe was your pain from your psoriasis during the past 24 h?

- None
- Mild
- Moderate
- Severe
- Very severe

2. How severe was the redness from your psoriasis during the past 24 h?

- None
- Mild
- Moderate
- Severe
- Very severe

3. How severe was your itching from your psoriasis during the past 24 h?

- None
- Mild
- Moderate
- Severe
- Very severe

4. How severe was your burning from your psoriasis during the past 24 h?

- None
- Mild
- Moderate
- Severe
- Very severe

© 2016, Boehringer Ingelheim International GmbH.

## Abbreviations

FDA: Food and Drug Administration
HIPPA: Health Insurance Portability and Accountability Act
IRB: Institutional Review Board
PRO: Patient-reported Outcomes
PSD: Psoriasis Symptom Diary
PSI: Psoriasis Symptom Index
PSS: Psoriasis Symptom Scale
